# Effects of Social Housing on Dairy Calf Social Bonding

**DOI:** 10.3390/ani12070821

**Published:** 2022-03-24

**Authors:** Emily E. Lindner, Katie N. Gingerich, Katharine C. Burke, Samantha B. Doyle, Emily K. Miller-Cushon

**Affiliations:** Department of Animal Sciences, University of Florida, Gainesville, FL 32611, USA; lindneremilye@ufl.edu (E.E.L.); kgingerich@ufl.edu (K.N.G.); burke.katharine@ufl.edu (K.C.B.); samantha.doyle@ufl.edu (S.B.D.)

**Keywords:** social housing, social bonding, social preference

## Abstract

**Simple Summary:**

Although cows and calves are typically separated at birth in intensive dairy farming, socially housing dairy calves provides an opportunity for social bonding. When tested in our study, calves housed in pairs preferred to be near their pen-mate, but spent more time near other calves regardless of their familiarity, compared to individually housed calves. In contrast, individually housed calves showed no preference between a calf housed within visual contact and a calf housed elsewhere in the barn. Additionally, the behavior of individually housed calves was affected by the first calf approached during the social preference test.

**Abstract:**

Social housing for dairy calves has a range of benefits for social development, yet there is limited understanding of how social bonds form early in life. We characterized effects of early life social contact on the development of social preference for calves varying in familiarity. A total of 40 calves were tested in a social preference test at 4 weeks of age to assess the formation of social bonds and preference for their peers. Within an open-field social preference test, focal calves were presented with two stimulus calves, one ‘more familiar’ and one ‘less familiar’. We found that pair-housed calves spent more time in close proximity with either stimulus calf and had a greater preference for their pen-mate, compared to another calf reared within visual contact. Individually housed calves exhibited no preference for calves reared within visual but not physical contact compared to calves that were completely unfamiliar. Of the calves that approached both stimulus calves, individually housed calves that approached the ‘less familiar’ calf first spent less time near the ‘more familiar’ calf, whereas behavior of pair-housed calves was not affected by the first calf approached. These results suggest that physical contact is necessary for the development of social bonds in young dairy calves, and early life social housing may support the development of normal social behavior in dairy cattle.

## 1. Introduction

Across species, physical interactions between mother-infant dyads have played an important role in bond formation among social animals. Dairy cattle are unique among intensively management farm animals in that the young are routinely raised without maternal contact, which removes a primary social bond in early life [1]. Although Holstein Friesian cattle were selected for the ability to cope with early calf separation and for reduced maternal behavior [2], the process of domestication has mostly led to an increase in pro-social behaviors [3]. Despite the importance of supporting formation of social bonds, individual housing remains common when rearing dairy calves (e.g., 63% of calves in the United States [4], 60% in Europe [5]). However, there are considerable benefits related to calf performance and social development associated with social housing. Calves reared with social contact, whether housed in pairs or larger groups, display more social behaviors, such as allogrooming and locomotor play following grouping [6,7], compared to calves reared individually. Additionally, calves reared with social contact experience performance benefits including greater solid feed intake during the pre-weaning period [8,9,10] and greater body weights following weaning [11,12]. Early social development may have critical implications in reduction of fearfulness, given evidence that socially reared calves have lower reactivity to novel environments [13] and stimuli, including feed [14].

Although physical contact between dairy cattle dams and their offspring is typically prevented, bonds with peers strengthen during the weaning period, based on evidence of preferential and prolonged social contact [15]. Furthermore, evidence suggests that social housing of dairy calves may support the development of social bonds with peers, with pair-housed calves engaging in social licking as early as 2 days of age [7]. In the absence of maternal contact, physical contact with same aged peers may therefore serve an important role in promoting social bonds, and therefore normal social development in calves.

In group-housed calves, there is evidence of preference for pen-mates that have been grouped together longer and from a younger age [16], suggesting that familiarity plays a role in social bonding. Calves observed in a semi-natural setting spent more time grazing and resting with certain individuals over the course of a six-month observation period [17], suggesting the formation of preferential bonds. These effects of familiarity on bonding can additionally be interpreted through responses in behavioral tests, as dairy calves choose to spend more time in proximity to a familiar calf following grouping, when tested in a y-maze [18]. Similarly, older heifers and cows choose to spend more time in close proximity to a familiar cow following regrouping [19]. More affiliative behaviors are also seen among calves and dams that are familiar with each other [9,20,21]. The increase in pro-social behaviors between known peers can be affected by the type of housing and amount of contact a calf is provided. Calves provided with full social contact were found to lick and sniff a familiar, companion calf more than an unfamiliar calf during a social preference test, versus calves provided limited contact through pen bars only, suggesting that social bonds are stronger between calves that have been housed together and for longer periods of time [22].

Despite evidence of increased social proximity between familiar pen-mates in group-housed calves, there has been limited exploration of the early life development of social preference in socially housed calves. Furthermore, despite frequent reference to the importance of maintaining visual contact with conspecifics in individually housed animals [23], the potential influence of this more limited contact on social bonding is unclear. Therefore, the objective of this experiment was to assess the effects of social housing, with calves housed individually or in pairs, on social preference for surrounding conspecifics. We explored social preference in an open arena test, where calves were presented with stimulus calves varying in familiarity, based on relative location within the barn. We hypothesized that calves that were pair-housed would prefer to spend more time near their pen-mate compared to a calf from an adjacent pen, whereas individually housed calves would express a preference for a calf from the adjacent pen compared to a calf housed elsewhere in the barn. We additionally assessed performance outcomes in early life, including feed intake, growth, and health, predicting that our results would support previous findings describing a beneficial effect of social contact on starter intake, leading to greater weight gain through weaning, with comparable health to individually housed calves.

## 2. Materials and Methods

### 2.1. Animals, Management, and Experiment Design

Holstein heifers (*n* = 40 focal calves; 60 calves total) were enrolled at the University of Florida Dairy Unit (Hague, FL, USA) during the months of September 2020 through May 2021. Calves were managed under standard operating procedures for the facility and all procedures were reviewed and approved by the University of Florida Institutional Animal Care and Use Committee (protocol #201910617). Calves were removed from their dams following birth, receiving 4 L of quality-controlled colostrum [checked using a digital refractometer, total solids > 25%] by bottle within 1 h of birth and were uniquely identified with radiofrequency identification ear tags.

Within 48 h following birth, calves were alternately assigned to either individual housing (*n* = 20 calves; birthweight 40.6 ± 5.1 kg) or pair housing (*n* = 20 pairs; 1 focal calf per pair; focal calf birthweight 39.9 ± 3.4 kg) for the first eight weeks of life. Sample size was based on previous work with similar response variables (effects of social contact on social preference; [18,22]). Pens were constructed of wire mesh with pens for pair-housed calves twice the size of individual calf pens (1.8 by 1.8 m, vs. 0.9 by 1.8 m). Paired and individual pens were alternated within the barn and the distance between adjacent pens was equal to the width of one pen (0.9 m) and prevented physical contact between calves in different pens. Pens were bedded with sand that was replaced when soiled, or otherwise on a weekly basis. Calves were provided 6 L/d of milk replacer (28% CP and 20% fat; Suwannee Valley Feeds; 150 g/L) in two feedings via teat bucket (Peach Teat Limited, Christchurch, New Zealand) at 0630 and 1500, until they were able to consume the entire allotment of milk, at which point they were provided 8 L/d in two feedings. Calves also had *ad libitum* access to water and pelleted calf starter (22% CP and 2% fat; Ampli-Calf Starter Warm Weather, Purina Animal Nutrition LLC, Shoreview, MN, USA) from birth. All pens were located in an open-sided barn providing protection from wind and rain. Calves were monitored daily by farm staff and research personnel.

Calves were disbudded by hot iron, using local anesthetic and analgesic as per facility protocols, following behavioral testing (at 31.4 ± 2.4 d of age, mean ± SD). At the beginning of week 6 of life (42.2 ± 1.9 d of age, mean ± SD), calves were weaned over a 10 d step down plan, consistent with standard farm operating procedure, receiving 3 L of milk for 4 days, 2 L for 4 days, and 1 L for 2 days to be completely weaned by the middle of week 7 of life (51.2 ± 1.9 d of age).

### 2.2. Performance Data Collection

For the first 8 weeks of life, milk and solid feed intake, daily health scores, and weekly weights were recorded. Milk intake was recorded each weekday during the morning feedings only, based on amount offered and amount refused. Daily starter intake was calculated at the pen level based on amount offered and amount refused.

Research personnel recorded daily rectal temperature and incidence of scours. Calves were recorded as scouring (binary outcome; 0 = no occurrence of scouring, 1 = scouring) if their stool was recorded as a score of 2 or 3, based on the Wisconsin Calf Health Scoring chart [24]. If any calf in a pen was scouring, oral electrolytes were added to one of the water sources in the pen.

Calves were weighed at birth and then weekly until 9 weeks of age.

### 2.3. Behavioral Testing

At approximately 4 weeks of age (29.3 ± 1.9 d of age, mean ± SD) calves were tested in a social preference test, conducted in an open arena, and modeled after other simple tests used to assess social preferences [18,22]. Individually housed calves (*n* = 20) and the focal calf from each pair (*n* = 20) were tested. Each calf was moved individually the short distance to the testing arena within a single wire mesh pen (0.9 by 1.8 m), where study personnel would slowly lift and carry the pen while the calf walked within it, a procedure to which calves were previously exposed to during routine husbandry. The focal and non-focal calves in each pair were moved individually to the testing arena. The testing arena (7 × 13 m) was bedded with sand and all four walls were covered with a dark green tarp to eliminate visual contact outside the arena during testing. The focal calf was initially exposed to the testing arena for 5 min to habituate to the new surroundings and decrease the effects of the new environment on their responses during the social preference test. After the 5 min had elapsed, the focal calf was removed from the arena, placed back into the individual pen, and moved to the side of the testing arena out of sight of the entrance.

**Figure 1:**
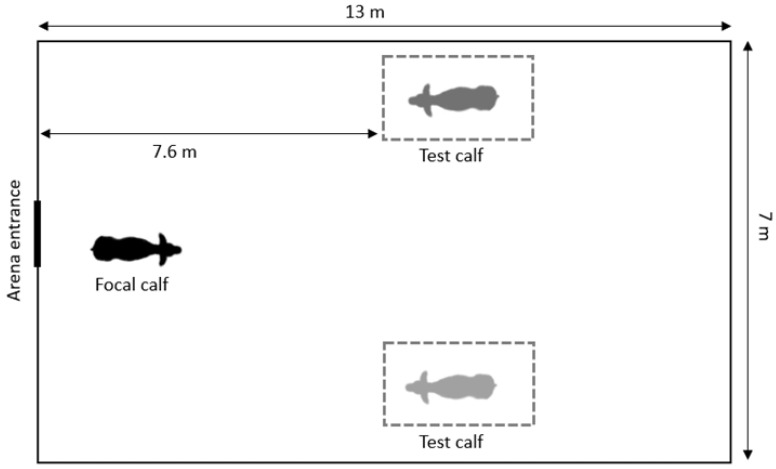
Layout of the social preference test within the testing arena, showing the placement of the stimulus calves (the ‘more familiar’ and ‘less familiar’ calves; side randomized) relative to the en2.4. Behavioral Observations and Calculations.

Directly following the habituation period, the social preference test was performed. Calves for the social preference test were selected based on their location relative to the focal calf in the main barn. Each calf was presented with a calf they were most familiar with (‘more familiar’ stimulus calf) and a calf that was a degree less familiar (‘less familiar’ stimulus calf), to explore the relative importance of physical contact vs. visual contact in the establishment of social bonds. For pair-housed calves, the non-focal pair calf was used as the ‘more familiar’ stimulus calf and the adjacent individually housed calf was used as the ‘less familiar’ stimulus calf. For individually housed calves, a calf from the adjacent pen was used as the ‘more familiar’ stimulus calf (either the neighboring non-focal pair calf or an adjacent individual calf) and a calf from elsewhere in the barn was used as the ‘less familiar’ stimulus calf. Calves used as stimuli in the social preference test remained in the individual wire mesh pens (0.9 by 1.8 m), which were placed on opposite sides of the arena, 7.6 m from the entrance (Figure 1). We randomized the placement of each of the stimulus calves to account for the effects of side bias and laterality effects, which are seen in mature cattle when contacting novel objects [25], and in other species regarding direction and handedness (dogs: [26]; cats: [27]). Following movement to their position within the arena, stimulus calves were allowed approximately 5 min to habituate to their surroundings. Study personnel observed the behavior of stimulus calves to ensure they were not unusually reactive (e.g., vocalizations, locomotor play) prior to commencing the test. Once the stimulus calves were placed on their respective sides, the focal calf was brought back to the arena. On the walk back to the arena entrance, the focal calf was turned twice 360 degrees to reduce the possibility of the calf going immediately to a known area in the arena. The gates of the arena were positioned to align with the center of the arena to avoid directing the calf toward either side. The individual pen used to move the calf was lifted to allow the calf to enter the arena. The calf remained in the arena for 5 min for the preference test.

Each test was recorded by video camera (GoPro Hero6, GoPro Inc., San Mateo, CA, USA). Behavioral responses were recorded continuously from video using Behavioral Observation Research Interactive Software (BORIS; [28]), according to the ethogram in Table 1, with the direction of all behaviors (toward the ‘more familiar’ or ‘less familiar’ calf noted). Behaviors were recorded by a single observer who was partially blind to treatments (we could not rule out possible recognition of calves from on-farm data collection) with an intra-observer reliability ≥ 0.90 (calculated within BORIS).

For each calf, we calculated total durations of calf-directed behavior (attention direction, contact, close proximity, and far proximity toward either stimulus calf in the arena), as well as the frequency of switching between stimulus calves, based on the ‘close proximity’ threshold. We also calculated total duration of behavior directed toward the ‘more familiar’ calf (duration on the side of the ‘more familiar’ calf, directing attention, in contact, close proximity, and far proximity).

Finally, we calculated preference ratios for each behavioral measure (side, attention, contact, close proximity, and far proximity), as the duration of each behavior directed toward the ‘more familiar’ calf, divided by the total duration of that behavior directed toward either of the stimulus calves.

### 2.4. Calculations and Statistical Analysis

We first assessed effects of housing treatment on the total duration of behavior directed toward either stimulus calf in the social preference test (total duration of attention directed, contact, close proximity, and far proximity) as well as latency to approach either stimulus calf, and frequency of switching between stimulus calves. These outcomes were analyzed in a general linear mixed model (MIXED procedure of SAS, SAS v. 9.4, SAS Institute, Cary, NC, USA) including the fixed effects of treatment, season (fall or spring, during which data were collected), and ‘more familiar’ calf side (left or right, to account for potential effects of laterality). Effects of treatment on first calf approached (analyzed as a binary outcome; 1 = calf approached the ‘more familiar’ calf first; 0 = calf approached the ‘less familiar’ calf first) were analyzed in a comparable model using a binary distribution (within Proc GLIMMIX).

Next, we analyzed behavior directed toward the ‘more familiar’ calf, in a model including the effects of housing treatment, first calf approached (‘more familiar’ or ‘less familiar’), and their interaction, as well as effects of season, and ‘more familiar’ calf side (left or right). For this evaluation of behavior directed toward the ‘more familiar’ calf, we included the effect of initial calf approached, after determining that not all calves approached both stimulus calves during the preference test. For this analysis, 2 calves were excluded as they never approached either stimulus calf.

Finally, we analyzed preference ratios within the social preference test. Given that some calves did not approach both stimulus calves, or remained within proximity of a stimulus calf for a short period of time (e.g., walking past without pausing), for this analysis of preference ratios, we set an inclusion criterion of calves that remained within close proximity of both stimulus calves for >6 s. This corresponded with eliminating the lowest 5% of values for duration of close proximity, resulting in the exclusion of a total of 11 calves (*n* = 6 PH and *n* = 5 IH). Effects of treatment on preference ratios were analyzed in a model including the fixed effects of treatment, season, and ‘more familiar’ calf side (left or right). We additionally tested whether preference ratios differed from 0.5 within each housing treatment using *t*-tests (TTEST procedure in SAS with the null hypothesis set at 0.5).

Early life performance data including milk intake, solid feed intake, rectal temperature, and ADG were summarized by week and analyzed using a general linear mixed model (within the MIXED procedure of SAS, v. 9.4, SAS Institute, Cary, NC, USA), with week as a repeated measure, fitted with the BIC covariance structure according to Schwarz’s Bayesian information criterion. Data were reported for individual calves, with the exception of solid feed intake, which was measured at the pen level, and was reported as kg/d/calf. The model included the fixed effect of week, treatment, week by treatment interaction, and the season. Days spent scouring were summarized for each calf and analyzed using a general linear mixed model and the model included the fixed effect of treatment.

All data were screened within Proc Univariate in SAS and model residuals were checked for normality, and some variables were square-root transformed (contact and far proximity) or log-transformed (latency to approach) to achieve normality. Back-transformed least square means and 95% confidence intervals were reported for data that were transformed for analysis. Significance was declared at *p* < 0.05 and trends were reported if 0.05 ≤ *p* ≤ 0.10.

## 3. Results

### 3.1. Effects of Housing Treatment on Total Duration of Calf-Directed Behavior

Pair-housed calves spent more time in ‘close proximity’ (<1 body length) with either stimulus calf (Table 2). Other behaviors, including attention directed, duration of contact, time spent in far proximity (between 1 and 2 body lengths of a stimulus calf), latency to approach, and the frequency of switching between calves did not differ between the housing treatments.

We found that all but two calves (2 IH) approached at least one of the stimulus calves (based on the ‘close proximity’ threshold).

### 3.2. Effects of the First Calf Approached and Housing Treatment on Duration of Behavior Directed toward the ‘More Familiar’ Calf

**Figure 2:**
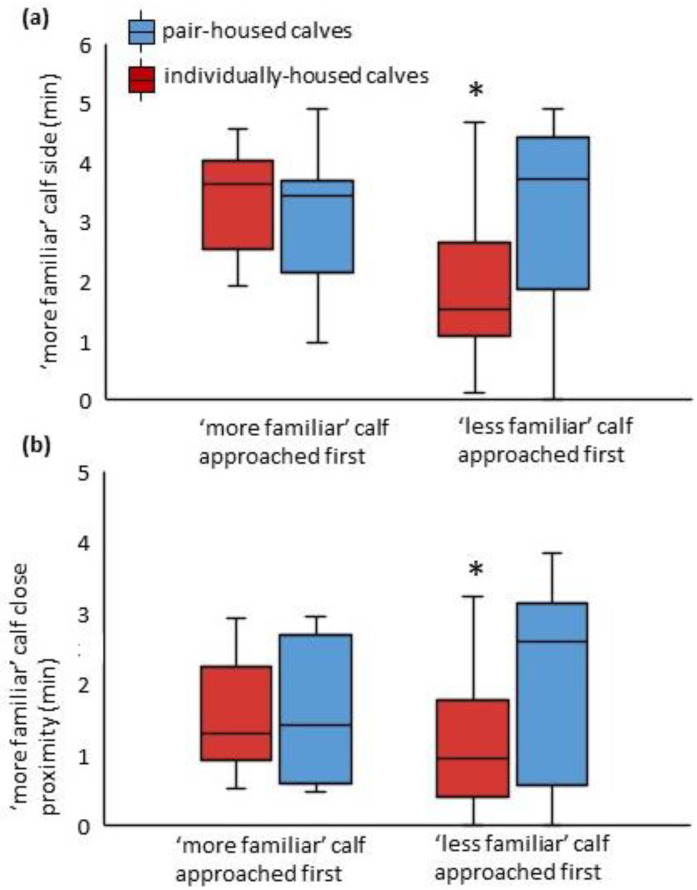
Effects of housing treatment and identity of first approached calf on duration of time spent on the side (**a**) or in close proximity (**b**) of the ‘more familiar’ calf, during a social preference test conducted at 4 weeks of age (at 29.3 ± 1.9 d of age). * Denotes significant pairwise difference between all other values (*p* < 0.05).

The first calf approached did not differ between treatments (54.3% vs. 34.6% of calves approached the ‘more familiar’ calf first; IH vs. PH; SE = 0.12; F_1,34_ = 1.21; *p* = 0.28), but we found that the identity of the initial approach calf influenced behavior within the preference test. The duration of behavior directed toward the ‘more familiar’ calf was subject to a interaction between housing treatment and identity of the first calf approached for side of the ‘more familiar’ calf (*p* = 0.049; Figure 2a) and in close proximity with the ‘more familiar’ calf (*p* = 0.090; Figure 2b). Individually housed calves that approached the ‘less familiar’ calf first spent less time on the side of the testing arena with the ‘more familiar’ calf, compared to individually housed calves that approached the ‘more familiar’ calf first, or pair-housed calves, regardless of their initial approach calf (Figure 2a). The same pattern was observed for duration of time spent in close proximity with the ‘more familiar calf’ (Figure 2b). In contrast, there was no interaction between housing treatment and initial approach calf affecting duration of time directing attention (overall mean: 0.56 min/calf; SE = 0.13; *p* = 0.47), in contact (overall mean: 0.11 min/calf; SE = 0.06; *p* = 0.20), and in far proximity (overall mean: 0.31 min/calf; SE = 0.08; *p* = 0.19) with the ‘more familiar’ calf. None of these outcomes were subject to an overall effect of initial approach calf (*p* > 0.14) or housing treatment (*p* > 0.25).

### 3.3. Effects of Housing Treatment on Social Preference Ratios When Both Stimulus Calves Were Approached

A fraction of calves did not approach both stimulus calves (5 IH calves; 4 approached the ‘less familiar’ calf, 1 approached the ‘more familiar’ calf only; and 6 PH calves, 3 approached the ‘less familiar’, 3 approached the ‘more familiar’ calf only). Therefore, we assessed effects of preference ratio only for those calves that spent time near both stimulus calves (using our inclusion criterion of >6 s in close proximity to both stimulus calves), we found that pair-housed calves had greater preference ratios for the side of the arena with the ‘more familiar’ calf and in close proximity with the ‘more familiar’ calf (Table 3). They additionally tended to have greater preference ratios for contact, and combined proximity and contact with the ‘more familiar’ calf. The ratio of time spent in attention-directed behaviors and far proximity did not differ between housing treatments (Table 3).

### 3.4. Effects of Social Housing on Calf Performance

**Figure 3:**
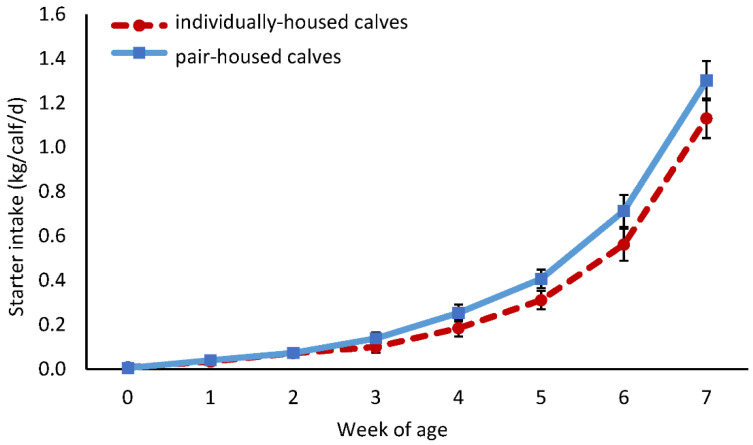
Starter intake (fresh weight) of calves housed individually (IH; *n* = 20) or in pairs (PH; *n* = 20 pairs) from birth to week 7 of age. Data were measured at the pen level and summarized by week. Error bars represent SE.

We saw no effect of housing treatment on milk intake from birth through weaning (3.3 vs. 3.3 L/meal, IH vs. PH, SE = 0.03, *p* = 0.72), with no interaction between treatment and week (*p* = 0.91). However, pair-housed calves tended to consume more solid feed from birth through weaning (0.30 vs. 0.37 kg/calf/d, IH vs. PH, SE = 0.03, *p* = 0.09), with an increase over time (*p* < 0.001) and no interaction between housing treatment and week (*p* = 0.69; Figure 3).

We found that ADG did not differ between housing treatments (0.53 vs. 0.58 kg/d, IH vs. PH, SE = 0.03, *p* = 0.20) with no interaction between treatment and week (*p* = 0.18). However, ADG changed between weeks (*p* < 0.001), particularly decreasing during weaning (0.064 vs. 0.32 kg/d; IH vs. PH; SE = 0.039). There were no differences in body weight between housing treatments (62.6 vs. 60.3 kg at end of study period; IH vs. PH, SE = 1.3, *p* = 0.60) with no interaction between treatment and week (*p* = 0.13).

Health outcomes were similar between treatments. Calf body temperature did not differ between housing treatment (38.6 vs. 38.7 °C, IH vs. PH, SE = 0.03, *p* = 0.83) and there was no interaction between treatment and week (*p* = 0.43). All calves scoured for at least 1 day, and the number of days spent scouring did not differ between housing treatments (6.4 vs. 5.6 d spent scouring; IH vs. PH; SE = 0.49, *p* = 0.29).

## 4. Discussion

The primary aim of this study was to assess the effects of early social contact on social preference for surrounding conspecifics, as assessed during a short preference test in a semi-novel environment. We predicted that calves that were provided with social contact would prefer to spend more time near their pen-mate compared to an adjacent calf, whereas individually housed calves would express a lesser preference for a calf from the adjacent pen compared to a calf housed elsewhere in the barn. In accordance with our hypothesis, calves that were provided with social contact from birth spent a greater amount of time near their pen-mate compared to an adjacent calf. However, we found that individually housed calves exhibited no preference for an adjacent calf, within visual but not physical contact, compared to an entirely unfamiliar calf, suggesting that physical social contact is critical in the formation of social bonds.

The stimulus calves presented in this preference test differed between housing treatments by design, given that degree of familiarity with surrounding calves differed between individually and pair-housed calves, providing insight into effects of only visual contact versus physical contact on the development of social bonds. Interestingly, we found no evidence to suggest that visual contact with the adjacent calf promoted any social preference for individually housed calves, compared to an unknown calf elsewhere in the barn. While heifers reared together can discriminate between familiar conspecifics [29], visual separation disrupts ability to recognize other calves [18], suggesting that olfactory and auditory cues alone may not be sufficient for social bond formation during development. Evidence in other species also suggests that physical contact may be necessary for social preferences to develop. For example, in nonhuman primates, physical contact plays an important role in the formation [30] and maintenance [31,32] of social bonds. Furthermore, evidence suggests that full social housing is more influential in development of social behavior, including display of affiliative behaviors (i.e., sniffing, allogrooming), than lesser degrees of social contact, such as head only contact [33] and transitions from limited to full contact (3 weeks isolated, 3 weeks full contact; [22]). In previous work, pair-housed calves also showed a preference for feeding alongside their pen-mate compared to feeding alone, whereas calves housed individually exhibited no preference for the adjacent calf, with which they had previously had limited visual and physical contact (through a window in the adjoining wall; [10]). While visual contact is often considered important to provide for livestock when physical contact in social housing is not possible [23], and may reduce stress; for example, visual contact with peers reduces behavioral reactivity during handling [34], and in a novel environment [35]. However, our results suggest that visual contact alone does not allow for the formation of social bonds between dairy calves.

We observed that pair-housed calves spent more total time in close proximity with either stimulus calf in the pen, suggesting a generally greater preference for social contact in a novel space. Previously, it was established that calves reared with social contact, compared to individually housed, engage in more social behavior following a regrouping event, perform more locomotor play with pen-mates [36], groom their peers more [37], and have longer durations of social rest, particularly in proximity with previous pen-mates [38]. This increased social ability or social comfort arising from social housing may explain why pair-housed calves spent more total time in close proximity with the stimulus calves, compared to individually housed calves. Furthermore, considering the testing context used in the present study, effects of housing treatment on social interaction with stimulus calves may depend on reduced fearfulness in novel environments previously noted in pair-housed calves [13], or improved ability to cope with stress [39,40]. Alternatively, this effect of housing treatment on total duration of calf-directed behavior may have been driven primarily by a specific preference for the pen-mate present in the arena, or have been enhanced by the greater level of familiarity with both stimulus calves in the pen, whereas individually housed calves only had some familiarity with one of the stimulus calves.

It is interesting to note that a fraction of calves did not interact with both test calves, suggesting that social motivations may compete with response to a novel environment. A longer habituation period may have influenced these responses. For example, in previous work, there was an effect of testing order on responses of group-housed calves exposed to repeated social separation tests in an unfamiliar pen, either alone, with an unfamiliar calf, or with a pen-mate [18]. Specifically, an interaction between testing order and calf familiarity was described, where test calves had a greater duration of contact with a familiar calf during the first test session, with no differences in subsequent test sessions. Differences in design in the present study, including a larger arena and a choice of social stimuli, may have yielded different outcomes over repeated testing. We suggest that greater habituation to the testing environment has the potential to either reduce degree of social contact with familiar calves (if familiar calves were approached for social support in an unfamiliar environment, as suggested by [18]) or increase social contact (as motivations shifted from exploration of the arena or, if previous fearful, calves became more willing to move throughout the space).

While the pen was open, allowing visual contact with both stimulus calves, we found no effect of treatment on initial choice within the social preference test. Previous evidence suggests that cattle are not adept at visually discriminating between conspecifics, even at shorter distances than in the present social preference test [41]. This appears to be the case in the current study, where latency to pass a close proximity threshold was not affected by identity of the stimulus calf, suggesting that initial choice may have been random. However, the identity of the first calf approached affected duration within close proximity for individually housed calves, with a reduction of time spent near the ‘more familiar’ calf if the ‘less familiar’ calf was approached first. In contrast, preference ratios of pair-housed calves were not sensitive to the first calf approached. In other studies, the initial choice in preference tests was found to affect behavioral responses including feeding behaviors [42,43], and mate choice [44]. In the present study, the interaction between treatment and effects of first calf approached could be attributed to a stronger social preference in pair-housed calves, as their behavior was less affected by initial calf encountered. Alternatively, this effect may be due to greater social exploration or awareness of the identity of both stimulus calves. Netser et al. [45] found that when mice were tested in a social preference test, they first exhibited an exploration phase with higher rates of transitions between stimuli, followed by an interaction phase with low transition rates and long interactions. In the present study, we found no effect of housing treatment on frequency of switching between test calves, but pair-housed calves did spend more total time in close proximity with either stimulus calf.

In the present study, we observed social contact and social proximity at two distance thresholds with the aim of obtaining a nuanced understanding of preference for conspecifics differing in degrees of familiar, based on physical or visual contact. Our findings generally suggest that duration of time within ‘close proximity’, measured here as less than one body length from another calf, may be a meaningful threshold for social interaction during short preference tests, given a range of effects of housing treatment on duration of time in this range. In contrast, duration of time below the ‘far proximity’ threshold (within 1–2 body lengths) was not affected in the present study, suggesting that this further proximity threshold, which was too far for physical touch, may be less reflective of social preference. We considered duration below different social proximity thresholds important to measure, considering that the size of the arena allowed the focal calf to easily be outside either range. Previous observation of weaned calves on a large pasture found evidence that duration of time spent within 1–3 body lengths of another calf decreased over time [46], suggesting that greater social proximity distances may be affected by calf age and environment.

We additionally assessed a range of important performance outcomes, with findings largely aligning with previous work and supporting performance benefits of social housing. We found that calves provided with social contact in the form of pairs tended to consume more solid feed compared to the individually housed calves from birth through weaning. This aligns with other research that supports the effects of social contact on increased solid feed intake in early life [10,47,48]. Additionally, we saw an effect of housing treatment on improved weight gain during weaning in pair-housed calves, coinciding with other studies [10,49,50]. In particular, we observed in the present study that weight gain during weaning was lower for individually housed calves, suggesting that they experienced a greater decrease in weight gain during weaning than pair-housed calves. Regarding calf health, concerns surrounding disease transmission are often cited as a motivator for individual housing; however, we saw no effect of housing treatment, calf body temperature or days spent scouring in the current study. Across the literature, the effects of social contact on health are variable, yet generally point to no relationship between social contact specifically and health outcomes [51].

## 5. Conclusions

Results of this social preference test conducted in an open arena suggest that dairy calves housed in pairs from birth are more social and have a preference for other calves reared in the same pen when tested in a social preference test. In contrast, individually housed calves exhibited no preference for a calf from a neighboring pen. Duration of behavior directed toward the ‘more familiar’ calf in the preference test was affected by the first calf approached for individually housed calves only, whereas social preferences of pair-housed calves were not sensitive to their initial social encounter. These results suggest that visual contact alone is not enough to form meaningful bonds in individually housed calves, whereas physical contact promotes social bonding in young pair-housed dairy calves.

## Figures and Tables

**Table 1:** Ethogram describing behaviors during the social preference test. Recipient calf (‘more familiar’ or ‘less familiar’ test calf) was noted for all behaviors.

Behavior	Description
Latency to approach	Time taken for focal calf to be within one body length of either stimulus calf, with the identity of first approached calf also noted.
Side of the arena	The side that the focal calf is on, based on the focal calf being on the left or right side of the arena; with each side noted as ‘more familiar’ or ‘less familiar’ side.
Attention directed	The focal calf’s head is directed toward a stimulus calf.
Contact	The focal calf makes direct contact with either their nose or mouth with a stimulus calf.
Close proximity	The focal calf is within one body length of a stimulus calf, but not physically touching the stimulus calf.
Far proximity	The focal calf is within two body lengths, and greater than one body length, of a stimulus calf.

**Table 2:** Effects of housing calves individually (IH; *n* = 20) or in pairs (PH; *n* = 20, one focal calf/pair) from birth on total duration of behavior directed toward either stimulus calf in a social preference test conducted at 4 weeks of age (29.3 ± 1.9 d of age).

Behavior ^1^	Treatment		SE	F_1,36_	*p*
	IH	PH			
Attention directed	1.0	1.1	0.09	0.01	0.91
Contact ^2,3^	0.21 (0.13, 0.31)	0.17 (0.11, 0.26)	-	0.37	0.55
Close proximity ^3^	2.2	3.0	0.23	5.38	0.03
Far proximity ^2,3^	0.51 (0.39, 0.65)	0.58 (0.45, 0.72)	-	0.51	0.48
Latency to approach ^2,4^	0.32 (0.18, 0.58)	0.21 (0.12, 0.37)	-	1.20	0.28
Frequency of switching between calves ^4^	5.1	4.0	0.70	1.05	0.31

^1^ All durations calculated as total behavior directed toward either calf, with units in minutes. ^2^ Contact and far proximity were square-root transformed, and latency to approach was log-transformed to meet assumptions of normality. Back-transformed least squares means and 95% CI are shown. ^3^ Contact is defined has the focal calf having direct contact with any part of the stimulus calf through the wire pen; close proximity defined as <1 body length of the stimulus calf, but not physically touching the stimulus calf; far proximity defined as >1, <2 body lengths of the stimulus calf. ^4^ Latency to approach and frequency of switching between calves was based on being within the close proximity threshold of the stimulus calf.

**Table 3:** Effects of housing treatment, with calves housed individually (IH) or in pairs (PH), on dairy calf ^1^ social preference ratios ^2^ during a social preference test conducted at 4 weeks of life (29.3 ± 1.9 d of age).

Preference Ratio	Treatment		SE	F_1,25_	*p*
	IH	PH			
Side	0.48	0.64 *	0.05	4.14	0.05
Attention directed	0.49	0.57	0.05	1.19	0.29
Contact ^3^	0.26 * (0.14, 0.43)	0.50 (0.32, 0.74)	-	3.60	0.07
Close proximity ^4^	0.47	0.67 *	0.06	4.40	0.05
Far proximity ^4^	0.53	0.59	0.06	0.49	0.49
Total proximity and contact ^5^	0.48	0.64 *	0.06	3.41	0.08

* *p* < 0.05, denotes that preference ratio differed significantly from 0.5. ^1^ Data reported for *n* = 15 IH calves and *n* = 14 PH calves, excluding calves that did not approach both stimulus calves (‘less familiar’ and ‘more familiar’ animals in the social preference test), based on a criteria of >6 s in close proximity with each animal during the test. ^2^ Calculated as duration of each behavior directed toward the ‘more familiar’ calf, divided by total duration of that behavior. ^3^ Contact was square-root transformed to meet assumptions of normality. Back-transformed least squares means and 95% CI are shown. ^4^ Close proximity defined as <1 body length of the stimulus calf, but not physically touching the stimulus calf; far proximity defined as >1, <2 body lengths of the stimulus calf, and any proximity is the summation of both proximity measures (total duration within two body lengths). ^5^ Total proximity and contact defined as the sum of close proximity (<1 body length of the stimulus calf), far proximity (>1, <2 body lengths of the stimulus calf), and direct physical contact with the stimulus calf.

## Data Availability

Data available in Supplementary Materials.

## Supplementary Materials

The following supporting information can be downloaded at https://www.mdpi.com/article/10.3390/ani12070821/s1, Supplementary Data S1: Raw data used for statistical analysis.

## References

[1] Newberry R.C., Swanson J.C. (2008). Implications of Breaking Mother-Young Social Bonds. Appl. Anim. Behav. Sci..

[2] Rørvang M.V., Nielsen B.L., Herskin M.S., Jensen M.B. (2018). Prepartum Maternal Behavior of Domesticated Cattle: A Comparison with Managed, Feral, and Wild Ungulates. Front. Vet. Sci..

[3] Clutton-Brock J. (1999). A Natural History of Domesticated Mammals.

[4] United States Department of Agriculture (2016). Dairy 2014, Part 1: Dairy Cattle Management Practices in the United States.

[5] Bolt S.L., Boyland N.K., Mlynski D.T., James R., Croft D.P. (2017). Pair Housing of Dairy Calves and Age at Pairing: Effects on Weaning Stress, Health, Production and Social Networks. PLoS ONE.

[6] Lv J., Zhao X.W., Su H., Wang Z.P., Wang C., Li J.H., Li X., Zhang R.X., Bao J. (2021). Effects of Group Size on the Behaviour, Heart Rate, Immunity, and Growth of Holstein Dairy Calves. Appl. Anim. Behav. Sci..

[7] Duve L.R., Jensen M.B. (2012). Social Behavior of Young Dairy Calves Housed with Limited or Full Social Contact with a Peer. J. Dairy Sci..

[8] Hepola H., Hänninen L., Pursiainen P., Tuure V.M., Syrjälä-Qvist L., Pyykkönen M., Saloniemi H. (2006). Feed Intake and Oral Behaviour of Dairy Calves Housed Individually or in Groups in Warm or Cold Buildings. Livest. Sci..

[9] Babu L.K., Pandey H.N., Sahoo A. (2004). Effect of Individual versus Group Rearing on Ethological and Physiological Responses of Crossbred Calves. Appl. Anim. Behav. Sci..

[10] Miller-Cushon E.K., DeVries T.J. (2016). Effect of Social Housing on the Development of Feeding Behavior and Social Feeding Preferences of Dairy Calves. J. Dairy Sci..

[11] Bernal-Rigoli J.C., Allen J.D., Marchello J.A., Cuneo S.P., Garcia S.R., Xie G., Hall L.W., Burrows C.D., Duff G.C. (2012). Effects of Housing and Feeding Systems on Performance of Neonatal Holstein Bull Calves. J. Anim. Sci..

[12] Costa J.H.C., Meagher R.K., von Keyserlingk M.A.G., Weary D.M. (2015). Early Pair Housing Increases Solid Feed Intake and Weight Gains in Dairy Calves. J. Dairy Sci..

[13] Jensen M.B., Vestergaard K.S., Krohn C.C., Munksgaard L. (1997). Effect of Single versus Group Housing and Space Allowance on Responses of Calves during Open-Field Tests. Appl. Anim. Behav. Sci..

[14] Costa J.H.C., Daros R.R., von Keyserlingk M.A.G., Weary D.M. (2014). Complesx social housing reduces food neophobia in dairy calves. J. Dairy Sci..

[15] Veissier I., Boissy A., Nowak R., Orgeur P., Poindron P. (1998). Ontogeny of Social Awareness in Domestic Herbivores. Appl. Anim. Behav. Sci..

[16] Raussi S., Niskanen S., Siivonen J., Hänninen L., Hepola H., Jauhiainen L., Veissier I. (2010). The Formation of Preferential Relationships at Early Age in Cattle. Behav. Processes.

[17] Reinhardt V., Mutiso F.M., Reinhardt A. (1978). Social Behaviour and Social Relationships between Female and Male Prepubertal Bovine Calves (Bos Indicus). Appl. Anim. Ethol..

[18] Færevik G., Jensen M.B., Bøe K.E. (2006). Dairy Calves Social Preferences and the Significance of a Companion Animal during Separation from the Group. Appl. Anim. Behav. Sci..

[19] Gutmann A.K., Špinka M., Winckler C. (2015). Long-Term Familiarity Creates Preferred Social Partners in Dairy Cows. Appl. Anim. Behav. Sci..

[20] Færevik G., Andersen I.L., Jensen M.B., Bøe K.E. (2007). Increased Group Size Reduces Conflicts and Strengthens the Preference for Familiar Group Mates after Regrouping of Weaned Dairy Calves (Bos Taurus). Appl. Anim. Behav. Sci..

[21] Val-Laillet D., Guesdon V., von Keyserlingk M.A.G., de Passillé A.M., Rushen J. (2009). Allogrooming in Cattle: Relationships between Social Preferences, Feeding Displacements and Social Dominance. Appl. Anim. Behav. Sci..

[22] Duve L.R., Jensen M.B. (2011). The Level of Social Contact Affects Social Behaviour in Pre-Weaned Dairy Calves. Appl. Anim. Behav. Sci..

[23] Federation of Animal Science Societies (2020). Guide for the Care and Use of Agricultural Animals in Research and Teaching.

[24] McGuirk S.M. (2008). Disease Management of Dairy Calves and Heifers. Vet. Clin. N. Am. Food Anim. Pract..

[25] Kappel S., Mendl M.T., Barrett D.C., Murrell J.C., Whay H.R. (2017). Lateralized Behaviour as Indicator of Affective State in Dairy Cows. PLoS ONE.

[26] Adámková J., Svoboda J., Benediktová K., Martini S., Nováková P., Tůma D., Kučerová M., Divišová M., Begall S., Hart V. (2017). Directional Preference in Dogs: Laterality and “Pull of the North”. PLoS ONE.

[27] McDowell L.J., Wells D.L., Hepper P.G. (2018). Lateralization of Spontaneous Behaviours in the Domestic Cat, Felis Silvestris. Anim. Behav..

[28] Friard O., Gamba M. (2016). BORIS: A Free, Versatile Open-Source Event-Logging Software for Video/Audio Coding and Live Observations. Methods Ecol. Evol..

[29] Hagen K., Broom D.M. (2003). Cattle Discriminate between Individual Familiar Herd Members in a Learning Experiment. Appl. Anim. Behav. Sci..

[30] Jablonski N.G. (2021). Social and Affective Touch in Primates and Its Role in the Evolution of Social Cohesion. Neuroscience.

[31] Majolo B., Schino G., Aureli F. (2012). The Relative Prevalence of Direct, Indirect and Generalized Reciprocity in Macaque Grooming Exchanges. Anim. Behav..

[32] Newton-Fisher N.E., Lee P.C. (2011). Grooming Reciprocity in Wild Male Chimpanzees. Anim. Behav..

[33] Holm L., Jensen M.B., Jeppesen L.L. (2002). Calves’ Motivation for Access to Two Different Types of Social Contact Measured by Operant Conditioning. Appl. Anim. Behav. Sci..

[34] Grignard L., Boissy A., Boivin X., Garel J.P., le Neindre P. (2000). The Social Environment Influences the Behavioural Responses of Beef Cattle to Handling. Appl. Anim. Behav. Sci..

[35] Boissy A., le Neindre P. (1990). Social Influences on the Reactivity of Heifers: Implications for Learning Abilities in Operant Conditioning. Appl. Anim. Behav. Sci..

[36] Zhang C., Juniper D.T., Meagher R.K. (2021). Effects of Physical Enrichment Items and Social Housing on Calves’ Growth, Behaviour and Response to Novelty. Appl. Anim. Behav. Sci..

[37] Jensen M.B., Larsen L.E. (2014). Effects of Level of Social Contact on Dairy Calf Behavior and Health. J. Dairy Sci..

[38] Lindner E.E., Gingerich K.N., Miller-Cushon E.K. (2021). Effects of Early Social Contact on Dairy Calf Response to Initial Social Grouping and Regrouping. J. Dairy Sci..

[39] Kikusui T., Winslow J.T., Mori Y. (2006). Social Buffering: Relief from Stress and Anxiety. Philos. Trans. R. Soc. B Biol. Sci..

[40] Raussi S., Lensink B.J., Boissy A., Pyykkönen M., Veissier I. (2003). The Effect of Contact with Conspecifics and Humans on Calves’ Behaviour and Stress Responses. Anim. Welf..

[41] Entsu S., Dohi H., Yamada A. (1992). Visual Acuity of Cattle Determined by the Method of Discrimination Learning. Appl. Anim. Behav. Sci..

[42] Huzzey J.M., Fregonesi J.A., von Keyserlingk M.A.G., Weary D.M. (2013). Sampling Behavior of Dairy Cattle: Effects of Variation in Dietary Energy Density on Behavior at the Feed Bunk. J. Dairy Sci..

[43] Meagher R.K., Weary D.M., von Keyserlingk M.A.G. (2017). Some like It Varied: Individual Differences in Preference for Feed Variety in Dairy Heifers. Appl. Anim. Behav. Sci..

[44] Reaney L.T. (2009). Female Preference for Male Phenotypic Traits in a Fiddler Crab: Do Females Use Absolute or Comparative Evaluation?. Anim. Behav..

[45] Netser S., Haskal S., Magalnik H., Wagner S. (2017). A Novel System for Tracking Social Preference Dynamics in Mice Reveals Sex- and Strain-Specific Characteristics. Mol. Autism.

[46] Horvath K.C., Miller-Cushon E.K. (2018). Characterizing Social Behavior, Activity, and Associations between Cognition and Behavior upon Social Grouping of Weaned Dairy Calves. J. Dairy Sci..

[47] Whalin L., Weary D.M., von Keyserlingk M.A.G. (2018). Pair Housing Dairy Calves in Modified Calf Hutches. J. Dairy Sci..

[48] Jensen M.B., Duve L.R., Weary D.M. (2015). Pair Housing and Enhanced Milk Allowance Increase Play Behavior and Improve Performance in Dairy Calves. J. Dairy Sci..

[49] Chua B., Coenen E., van Delen J., Weary D.M. (2002). Effects of Pair versus Individual Housing on the Behavior and Performance of Dairy Calves. J. Dairy Sci..

[50] Knauer W.A., Godden S.M., Rendahl A.K., Endres M.I., Crooker B.A. (2021). The Effect of Individual versus Pair Housing of Dairy Heifer Calves during the Preweaning Period on Measures of Health, Performance, and Behavior up to 16 Weeks of Age. J. Dairy Sci..

[51] Costa J.H.C., von Keyserlingk M.A.G., Weary D.M. (2016). Invited Review: Effects of Group Housing of Dairy Calves on Behavior, Cognition, Performance, and Health. J. Dairy Sci..

